# Selenopolysaccharide Isolated from *Lentinula edodes* Mycelium Affects Human T-Cell Function

**DOI:** 10.3390/ijms252111576

**Published:** 2024-10-28

**Authors:** Beata Kaleta, Katarzyna Zielniok, Aleksander Roszczyk, Jadwiga Turło, Radosław Zagożdżon

**Affiliations:** 1 Department of Clinical Immunology, Medical University of Warsaw, Nowogrodzka 59, 02-006 Warsaw, Poland; aleksander.roszczyk@gmail.com; 2 Laboratory of Cellular and Genetic Therapies, Center for Preclinical Research, Medical University of Warsaw, Banacha 1B, 02-097 Warsaw, Poland; radoslaw.zagozdzon@wum.edu.pl; 3 Department of Drug Technology and Pharmaceutical Biotechnology, Medical University of Warsaw, Banacha 1, 02-097 Warsaw, Poland; jadwiga.turlo@wum.edu.pl

**Keywords:** *Lentinula edodes*, T cells, immune checkpoints, cytokines, immunomodulation

## Abstract

*Lentinula edodes* polysaccharides are natural immunomodulators. SeLe30, analyzed in this study, is a new mixture of selenium-enriched linear 1,4-α-glucans and 1,3-β- and 1,6-β-glucans isolated from L. edodes mycelium. In the present study, we evaluated its immunomodulatory properties in human T cells. Peripheral blood mononuclear cells (PBMCs) and T cells were isolated from healthy donors’ buffy coats. The effects of SeLe30 on CD25, CD366, and CD279 expression, the subsets of CD8+ T cells, and IFN-γ, IL-6, and TNF-α production were analyzed. SeLe30 downregulated CD25, CD279, and CD366 expression on T cells stimulated by the anti-CD3 antibody (Ab) and upregulated in unstimulated and anti-CD3/CD28-Abs-stimulated T cells. It increased the percentage of central memory CD8+ T cells in unstimulated PBMCs and naïve and central memory T cells in anti-CD3-Ab-stimulated PBMCs. SeLe30 decreased the number of central memory and naïve CD8+ T cells in anti-CD3/CD28-stimulated T cells, whereas, in PBMCs, it reduced the percentage of effector memory CD8+ T cells. Moreover, SeLe30 upregulated cytokine production. SeLe30 exhibits context-dependent effects on T cells. It acts on unstimulated T cells, affecting their activation while increasing the expression of immune checkpoints, which sensitizes them to inhibitory signals that can silence this activation. In the case of a lack of costimulation, SeLe30 exhibits an inhibitory effect, reducing T-cell activation. In cells stimulated by dual signals, its effect is further enhanced, again increasing the “safety brake” of CD366 and CD279. However, the final SeLe30 effect is mediated by its indirect impacts by altering interactions with other immune cells.

## 1. Introduction

*Lentinula edodes* (Berk.) Pegl., more commonly known as Shiitake mushroom, is a source of numerous substances with immunomodulatory, anticancer, antioxidant, and antibacterial properties [1,2]. The most important bioactive components of *L. edodes* are polysaccharides: α- and β-glucans. Studies conducted on animal models demonstrated that these compounds have immunomodulatory properties [3]. They have been shown to increase the chemotactic activity of macrophages [4,5], nitric oxide (NO) production [6,7], and natural killer (NK) cell activity [8]. Moreover, research indicated that polysaccharides from *L. edodes* enhanced lymphocyte proliferation [7] and activation [8,9,10,11,12] and regulated cytokine synthesis, including interleukin (IL)-1β, IL-2, IL-4, IL-6, IL-10, and IL-12; interferon (IFN)-γ; and tumor necrosis factor (TNF)-α [5,7,9,10,11,13,14,15,16,17]. Studies on the biological activity of *L. edodes* polysaccharides in humans were initiated in the 1980s and concerned mainly lentinan: β-1,6: β-1,3-glucan isolated from *L. edodes* fruiting bodies [18,19,20,21]. This compound has been shown to increase the proliferation of human peripheral blood mononuclear cells (PBMCs), the number of T and B cells, the activity of NK cells, and the production of TNF-α, IL-1α and IL-1β, IL-10, IL-12, TGF-β, and IFN-γ [22,23,24]. Therefore, lentinan is clinically used as an adjuvant in anticancer therapies in China and Japan [25,26].

The exact immunomodulatory mechanism of action of fungal polysaccharides is not known, but it has been documented that these compounds have an affinity for numerous receptors located on human immunocompetent cells, including complement receptor type 3 (CR3), dectin-1, lactosyceramide (LaCer), scavenger, toll-like receptors (TLRs), and CD28 receptors [27,28,29,30,31,32]. It has also been confirmed that the biological activity of L. edodes polysaccharides depends on their source, the methods of cultivation, and extraction procedures, as these factors affect their composition, molecular weight, and conformation [3].

Selenium (Se) is a trace mineral and one of the most essential microelements for humans. It is worth emphasizing that Se is the only element whose incorporation into proteins is genetically encoded as the constitutive part of selenocysteine. Se is important for immune system and muscle function, reproductive biology, and the cardiovascular and central nervous systems [33]. Se regulates the function of almost all immune cells [34]. It has been demonstrated that Se increases immunoglobulin production by B cells, enhances differentiation and proliferation of T and B cells, and upregulates natural killer (NK) cells’ cytotoxic effects and neutrophils’ chemotaxis. Moreover, Se, due to its antioxidant and neuroprotective activities, plays a significant role in the prevention and treatment of Alzheimer’s and Parkinson’s diseases [35]. In addition, it has been found that Se is associated with reproductive function in men and women. In women, Se regulates the progression of granulosa cells, oocyte growth, and estradiol synthesis. In men, this microelement is essential for the production of sperm cells, as well as for the maturation of spermatozoa. Se deficiency has also been found to be associated with skeletal muscle disorders, thyroid disorders (such as autoimmune thyroiditis and Grave’s disease), and ovarian, pancreatic, and lung cancer [34,35]. As mentioned above, Se is incorporated into proteins in the form of selenocysteine. In humans, 25 selenoproteins, which play an important role in the regulation of the immune system, have been identified [36]. The most important immunomodulating selenoproteins are glutathione peroxidases, thioredoxin reductases, iodothyronine deiodinases, methionine-R-sulfoxide reductase B1, selenophosphate synthetase 2, and selenoprotein K. It has been demonstrated that selenoproteins play an essential role in anti-inflammatory responses and antioxidant processes [36,37,38].

In our previous investigations, we isolated a Se-containing polysaccharide fraction from the Se-enriched mycelium of *L. edodes* (named SeLe30). This fraction is a mixture of linear 1,4-α-glucan and linear 1,3-β- and 1,6-β-glucans with a high molecular weight (3.62 × 10^6^ g/mol) [39]. Preliminary studies of the biological activity of SeLe30 have shown that it significantly downregulated the proliferation of PBMCs stimulated with an anti-CD3 antibody (Ab) and allostimulated in a mixed lymphocyte reaction, and it decreased the production of intracellular TNF-α by CD3+ T cells [40,41,42]. In the next study, we focused on assessing the effects of SeLe30 on the activation, proliferation, and cytokine production of two populations of human T cells: CD4+ and CD8+ cells. Additionally, the effect of SeLe30 on T cells was compared using two different types of activation (anti-CD3 and anti-CD3/CD28 Abs) [43]. It was shown that the effect depends on the type of T-cell stimulation. The polysaccharide inhibited the activation, proliferation, and production of IL-2 and IL-4 by anti-CD3-stimulated T cells, which confirmed our previous observations. However, SeLe30 enhanced T-cell activation and increased the number of divided T cells and IFN-γ production in anti-CD3/CD28-stimulated cells. Interestingly, we reported that, regardless of the stimulation used, SeLe30 upregulated the production of IL-6 and IL-10, which suggested its effect also on peripheral blood monocytes, which could be present in small amounts in the cultures. To exclude the contribution of other immune cells to the effects of SeLe30 on T cells, in the present study, we performed analyses on CD3+ T cells isolated from PBMCs and then compared the effects exerted on PBMCs. We assessed the influence of SeLe30 on the expression of the activation marker CD25 and immune system-controlling molecules: CD366 (also known as T-cell immunoglobulin and mucin-containing molecule 3 (TIM-3)) and CD279 (also known as programmed cell death protein 1 (PD-1)). Moreover, we investigated the effect of SeLe30 on the proportions of subsets of CD8+ T cells and cytokine production.

As *L. edodes* polysaccharides, α- and β-glucans are valuable functional substances distributed in the cell walls of fungi. Their unique features have made them broadly applied in medicine. *L. edodes* polysaccharides are classified as biological response modifiers (BRMs) and, therefore, are attractive candidates for use in the treatment of inflammatory and infectious diseases and cancers.

## 2. Results

### 2.1. The Effect of SeLe30 on the Expression of ICPs and Activation Markers on T Cells

**Figure 1:**
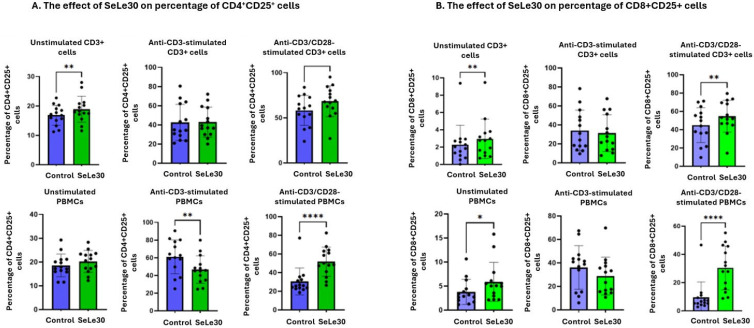
The effect of SeLe30 on the percentage of CD4+CD25+ cells (**A**) and CD8+CD25+ cells (**B**) in isolated CD3+ T cells (top row) or PBMCs (bottom row). CD3+ T cells or PBMCs isolated from healthy donors’ buffy coats were unstimulated or stimulated with anti-CD3 Ab or anti-CD3/CD28 Abs and incubated in the presence of 100 µg/mL of SeLe30 for 24 h. The mean values *(n* = 22) and standard deviations are given. * *p* < 0.05; ** *p* < 0.01; **** *p* < 0.0001.

The effect of SeLe30 on the expression of the activation marker CD25 on T cells is presented in Figure 1.

It was demonstrated that SeLe30 increased the percentage of CD4+CD25+ and CD8^+^CD25^+^ in unstimulated isolated CD3+ T cells (*p* = 0.0035 and *p* = 0.0032, respectively). Conversely, in the case of unstimulated PBMC cultures, this compound decreased the percentage of CD8+CD25+ cells (*p* = 0.0464) and did not affect the number of CD4+CD25+ cells. When isolated CD3+ T cells were stimulated by anti-CD3 Ab, no effect of the polysaccharide on changes in CD25 marker expression was demonstrated. However, SeLe30 decreased the percentage of CD4+CD25+ cells in PBMC cultures (*p* = 0.0017) and had no effect on CD8+ cells. When isolated CD3+ T cells were stimulated with a double signal (anti-CD3/CD28 Abs), an increase in the percentage of CD4+ and CD8+ cells expressing CD25 was observed (*p* = 0.0041 and *p* = 0.0066, respectively). Similar results were obtained in PBMC cultures, but here, the effect of SeLe30 was stronger for both populations (*p* < 0.0001 for CD4+CD25+ and *p* < 0.0001 for CD8+CD25+).

**Figure 2:**
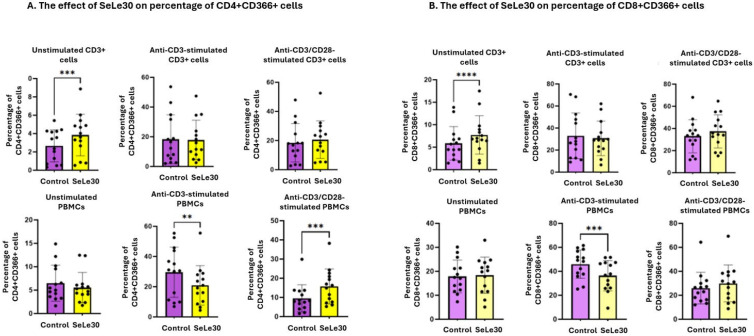
The effect of SeLe30 on the percentage of CD4+CD366+ cells (**A**) and CD8+CD366+ cells (**B**) in isolated CD3+ T cells (top row) or PBMCs (bottom row). CD3+ T cells or PBMCs isolated from healthy donors’ buffy coats were unstimulated or stimulated with anti-CD3 Ab or anti-CD3/CD28 Abs and incubated in the presence of 100 µg/mL of SeLe30 for 24 h. The mean values (*n* = 22) and standard deviations are given. ** *p* < 0.01; *** *p* < 0.001; **** *p* < 0.0001.

The effect of SeLe30 on the percentage of T cells expressing CD366 (TIM-3) is presented in Figure 2.

It was demonstrated that SeLe30 increased the percentage of CD4+CD366+ and CD8+CD366+ in unstimulated isolated CD3+ T cells (*p* = 0.0008 and *p* < 0.0001, respectively). In contrast, for unstimulated PBMC cultures, a similar effect was not observed. When isolated CD3+ T cells were stimulated by anti-CD3 Ab, SeLe30 did not affect the number of CD366-positive CD4+ and CD8+ T cells. However, SeLe30 significantly decreased the percentage of CD4+CD366+ T cells and CD8+CD366+ T cells in PBMC cultures (*p* = 0.0060 and *p* = 0.0001, respectively). When isolated CD3+ T cells were stimulated with anti-CD3/CD28 Abs, SeLe30 did not change the percentage of CD4+ and CD8+ cells expressing the CD366 marker. However, in PBMC cultures, the polysaccharide increased the number of CD4+CD366+ T cells (*p* = 0.0001), with no impact on CD8+ T cells.

**Figure 3:**
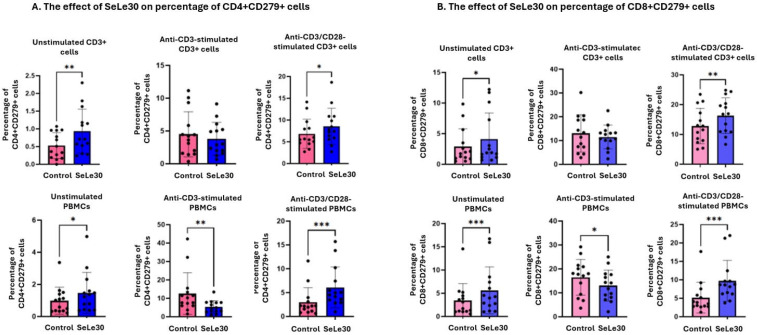
The effect of SeLe30 on the percentage of CD4+CD279+ cells (**A**) and CD8+CD279+ cells (**B**) in isolated CD3+ T cells (top row) or PBMCs (bottom row). CD3+ T cells or PBMCs isolated from healthy donors’ buffy coats were unstimulated or stimulated with anti-CD3 Ab or anti-CD3/CD28 Abs and incubated in the presence of 100 µg/mL of SeLe30 for 24 h. The mean values (*n* = 22) and standard deviations are given. * *p* < 0.05; ** *p* < 0.01; *** *p* < 0.001.

The effect of SeLe30 on the percentage of T cells expressing CD279 (PD-1) is presented in Figure 3.

SeLe30 was shown to increase the percentage of CD4+CD279+ and CD8+CD279+ in unstimulated isolated CD3+ T cells (*p* = 0.0060 and *p* = 0.0295, respectively), as well as in unstimulated PBMCs (*p* = 0.0366 and *p* = 0.0001, respectively). When isolated CD3+ T cells were stimulated by anti-CD3 Ab, SeLe30 did not affect the number of CD279-positive CD4+ and CD8+ T cells. However, SeLe30 decreased the percentage of CD4+CD279+ T cells and CD8+CD279+ T cells in PBMC cultures (*p* = 0.0012 and *p* = 0.0460, respectively). When isolated CD3+ T cells were stimulated with anti-CD3/CD28 Abs, SeLe30 increased the number of CD4+CD279+ T cells and CD8+CD279+ T cells (*p* = 0.0211 and *p* = 0.0017, respectively). Similar results were observed in PBMC cultures (*p* = 0.0001 for both populations).

### 2.2. The Effect of SeLe30 on CD8 T-Cell Subsets

**Figure 4:**
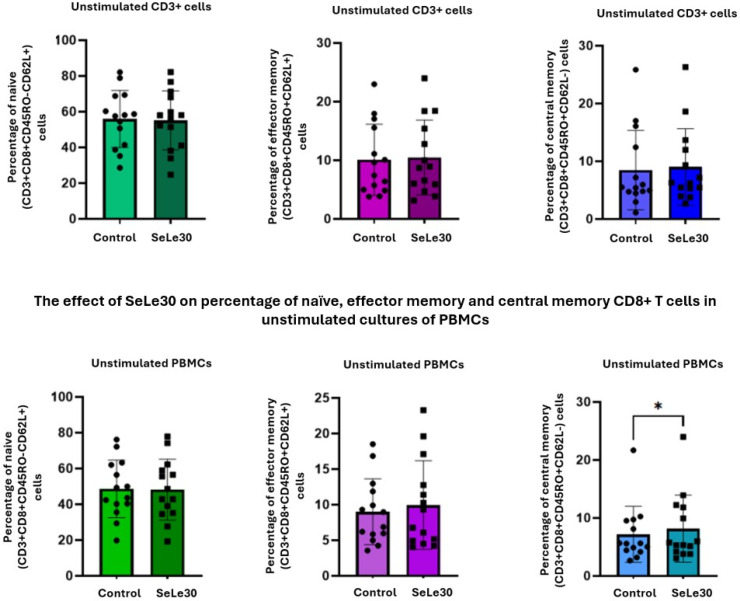
The effect of SeLe30 on the percentage of naïve, effector memory, and central memory CD8+ T cells in unstimulated cultures of isolated CD3+ T cells (top row) or PBMCs (bottom row). CD3+ T cells or PBMCs isolated from healthy donors’ buffy coats were unstimulated or stimulated with anti-CD3 Ab or anti-CD3/CD28 Abs and incubated in the presence of 100 µg/mL of SeLe30 for 24 h. The mean values (*n* = 22) and standard deviations are given. * *p* < 0.05.

**Figure 5:**
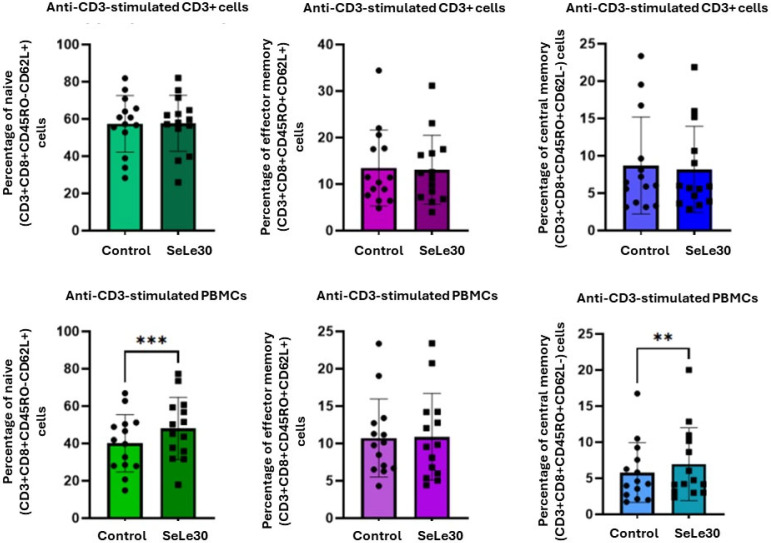
The effect of SeLe30 on the percentage of naïve, effector memory, and central memory CD8+ T cells in anti-CD3 stimulated cultures of isolated CD3+ T cells (top row) or PBMCs (bottom row). CD3+ T cells or PBMCs isolated from healthy donors’ buffy coats were unstimulated or stimulated with anti-CD3 Ab or anti-CD3/CD28 Abs and incubated in the presence of 100 µg/mL of SeLe30 for 24 h. The mean values (*n* = 22) and standard deviations are given. ** *p* < 0.01; *** *p* < 0.001.

**Figure 6:**
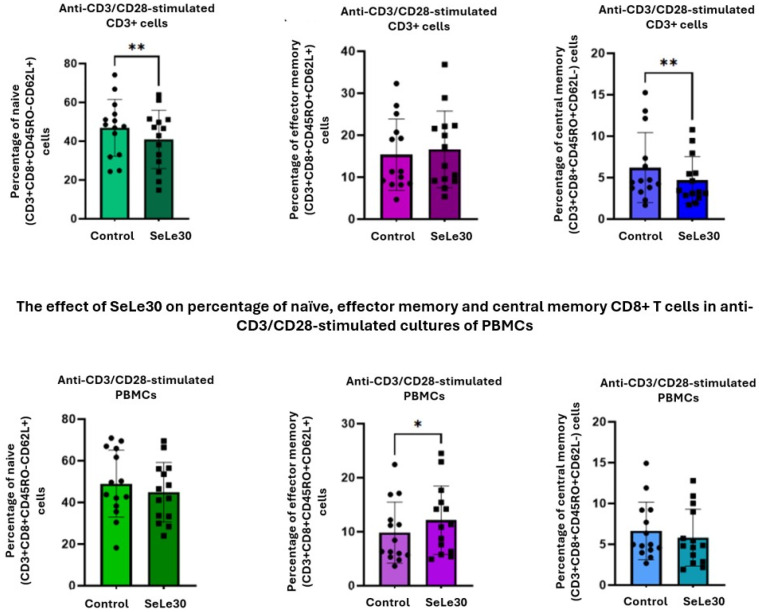
The effect of SeLe30 on the percentage of naïve, effector memory, and central memory CD8+ T cells in anti-CD3/CD28-stimulated cultures of isolated CD3+ T cells (top row) or PBMCs (bottom row). CD3+ T cells or PBMCs isolated from healthy donors’ buffy coats were unstimulated or stimulated with anti-CD3 Ab or anti-CD3/CD28 Abs and incubated in the presence of 100 µg/mL of SeLe30 for 24 h. The mean values (*n* = 22) and standard deviations are given. * *p* < 0.05; ** *p* < 0.01.

The effect of SeLe30 on the populations of CD8+ naïve, effector, and central memory CD8+ T cells is presented in Figure 4 (unstimulated cells), Figure 5 (cells stimulated with anti-CD3 Ab), and Figure 6 (cells stimulated with anti-CD3/CD28 Abs).

In unstimulated PBMC cultures, SeLe30 was shown to significantly increase the percentage of central memory CD8+ T cells (CD3+CD8+CD45RO+CD62L−) (*p* = 0.0203), but no effect was observed on other CD8+ T-cell subsets. SeLe30 did not affect the percentage of CD8+ T-cell subpopulations upon stimulation with anti-CD3 Ab in cultures with isolated CD3+ T cells. However, it was shown that SeLe30 significantly increased the percentage of naïve (CD3+CD8+CD45RO−CD62L+) and central memory (CD3+CD8+CD45RO+CD62L−) T cells (*p* = 0.0006 and *p* = 0.0085, respectively) in PBMC cultures stimulated with anti-CD3 Ab. When isolated CD3+ T cells were stimulated with a double signal (anti-CD3/CD28 Abs), SeLe30 decreased the percentage of central memory (CD3+CD8+CD45RO+CD62L−) and naïve (CD3+CD8+CD45RO−CD62L+) CD8+ T cells (*p* = 0.0031 and *p* = 0.0011, respectively), whereas, in PBMC cultures, it reduced the percentage of effector memory (CD3+CD8+CD45RO+CD62L+) CD8 T cells (*p* = 0.0295).

### 2.3. The Effect of SeLe30 on Cytokine Production in T Cells

**Figure 7:**
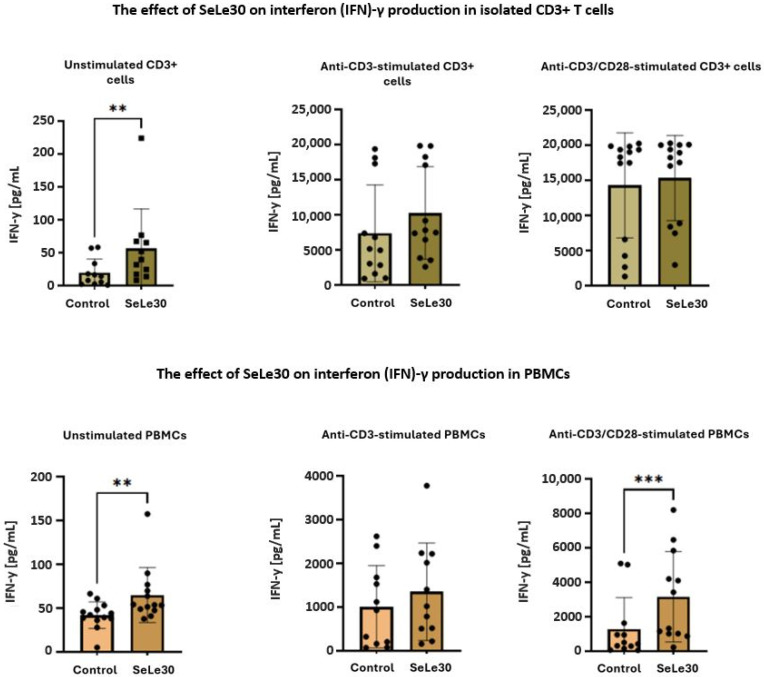
The effect of SeLe30 on interferon (IFN)-γ production in isolated CD3+ T cells (top row) or PBMCs (bottom row). CD3+ T cells or PBMCs isolated from healthy donors’ buffy coats were unstimulated or stimulated with anti-CD3 Ab or anti-CD3/CD28 Abs and incubated in the presence of 100 µg/mL of SeLe30 for 24 h. The mean values (*n* = 22) and standard deviations are given. ** *p* < 0.01; *** *p* < 0.001.

The effect of SeLe30 on the production of IFN-γ in T cells is presented in Figure 7.

It was shown that the polysaccharide increased the production of IFN-γ in both unstimulated cultures of isolated CD3+ T cells (19.57 ± 21.05 pg/mL vs. 56.32 ± 60.27 pg/mL, *p* = 0.0068) and PBMC cultures (41.96 ± 15.14 pg/mL vs. 64.99 pg/mL ± 31.32 pg/mL, *p* = 0.0046). However, SeLe30 did not affect IFN-γ production in cultures of isolated CD3+ T and PBMCs stimulated with anti-CD3 Ab. Conversely, SeLe30 significantly enhanced IFN-γ production in anti-CD3/CD28-Abs-stimulated PBMCs (1287.0 ± 1816 pg/mL vs. 3152.0 ± 2625 pg/mL, *p* = 0.0005). A similar effect was not observed in anti-CD3/CD28-Abs-stimulated CD3+ T cells.

**Figure 8:**
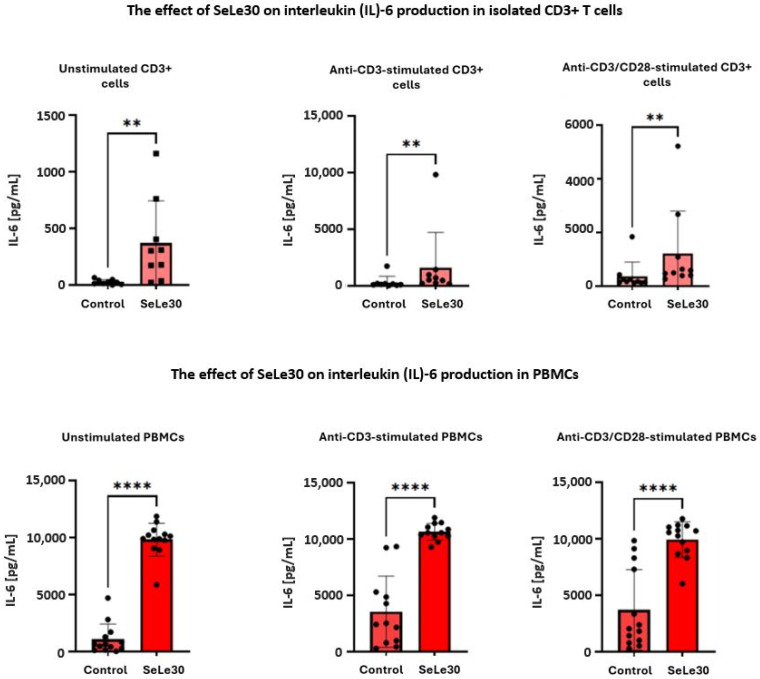
The effect of SeLe30 on interleukin (IL)-6 production in isolated CD3+ T cells (top row) or PBMCs (bottom row). CD3+ T cells or PBMCs isolated from healthy donors’ buffy coats were unstimulated or stimulated with anti-CD3 Ab or anti-CD3/CD28 Abs and incubated in the presence of 100 µg/mL of SeLe30 for 24 h. The mean values (*n* = 22) and standard deviations are given. ** *p* < 0.01; **** *p* < 0.0001.

The effect of SeLe30 on the production of IL-6 in T cells is presented in Figure 8.

It was demonstrated that the polysaccharide increased IL-6 production by unstimulated isolated CD3+ T cells (26.05 ± 21.21 pg/mL vs. 371.5 ± 370.0 pg/mL, *p* = 0.0039) and PBMCs (1072.0 ± 1338 pg/mL vs. 9821 ± 1441 pg/mL, *p* < 0.0001), anti-CD3 Ab-stimulated CD3+ T cells (303.3 ± 543.9 pg/mL vs. 1623.0 ± 3102 pg/mL, *p* = 0.0078) and PBMCs (3544 ± 3161 pg/mL vs. 10643 ± 743.7 pg/mL, *p* < 0.0001), and anti-CD3/CD28-stimulated CD3+ T cells 367.3 ± 527.0 pg/mL vs. 1223.0 ± 1570 pg/mL, *p* = 0.0078) and PBMCs (3703.0 ± 3559 pg/mL vs. 9926 ± 1580 pg/mL, *p* < 0.0001).

**Figure 9:**
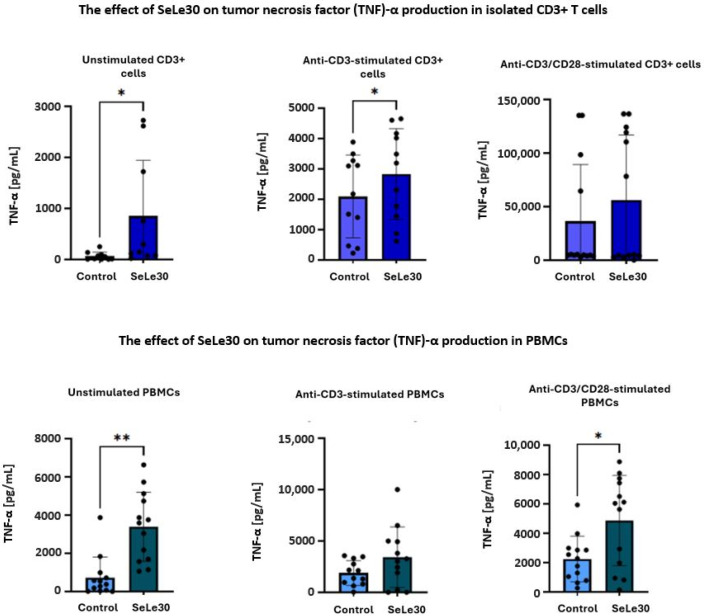
The effect of SeLe30 on tumor necrosis factor (TNF)-α production in isolated CD3+ T cells (top row) or PBMCs (bottom row). CD3+ T cells or PBMCs isolated from healthy donors’ buffy coats were unstimulated or stimulated with anti-CD3 Ab or anti-CD3/CD28 Abs and incubated in the presence of 100 µg/mL of SeLe30 for 24 h. The mean values (*n* = 22) and standard deviations are given. * *p* < 0.05; ** *p* < 0.01.

The effect of SeLe30 on the production of TNF-α in T cells is presented in Figure 9.

SeLe30 increased TNF-α production in both unstimulated isolated CD3+ T cells (65.35 ± 79.56 pg/mL vs. 855.0 ± 1087 pg/mL, *p* = 0.0384) and PBMCs (729.8 ± 1073 pg/mL vs. 3394 ± 1812 pg/mL, *p* = 0.0011). A similar effect was observed in cultures of CD3+ T cells stimulated with anti-CD3 Ab (2094.0 ± 1362 pg/mL vs. 2830.0 ± 1494 pg/mL, *p* = 0.0294), but not in PBMC cultures. In addition, SeLe30 upregulated TNF-α production in PBMCs stimulated with anti-CD3/CD28 Abs (2246 ± 1558 pg/mL vs. 4865.0 ± 3080 pg/mL, *p* = 0.0155), but not in isolated CD3+ T cells.

## 3. Discussion

Medicinal mushrooms, including *L. edodes*, are widely used, especially in Asian countries (e.g., China, Japan, Taiwan, and Korea), due to the presence of compounds with immunomodulatory, anti-inflammatory, and/or anticancer effects [1]. Over the past several years, multiple studies have identified various *L. edodes*-derived compounds responsible for the individual directions of pharmacological action [2]. The most important bioactive substances isolated from *L. edodes* are polysaccharides, mainly α- and β-glucans with proven immunomodulatory properties. It is worth emphasizing that the direction and strength of their biological activity depend on the structures of these compounds, particularly on the monosaccharide composition, solubility, and molecular weight, as well as their conformation [3,4]. The prospects for their clinical application are enhanced by the fact that isolated polysaccharide fractions subjected to bioengineering processes or chemical modifications of their structures (such as selenation) demonstrate improved therapeutic properties. Polysaccharides from *L. edodes* act as immune modulators, with the ability to induce various mechanisms of effector or tolerogenic immune responses. By modulating the initiation of the early innate immune response through direct action on monocytes/macrophages and innate lymphoid cells (ILCs) or by regulating the production of cytokines and chemokines, as well as the function of adaptive immune responses involving T and B cells, they enhance the effectiveness of immune system protection.

We have previously demonstrated that SeLe30 inhibited the proliferation of human T cells stimulated with anti-CD3 Ab and reduced intracellular TNF-α production by these cells, without an impact on B cells and granulocytes [40,41,42,43]. In further studies, we confirmed that this compound inhibited anti-CD3-stimulated T-cell activation, proliferation, and production of IL-2 and IL-4 but, in contrast, upregulated T-cell activation, the number of divided T cells, and IFN-γ production in cultures stimulated with anti-CD3/CD28 Abs. We performed these studies on PBMC cultures, as this model provided the best in vitro representation of SeLe30’s effects on human immune cells. Notably, regardless of the stimulation used and the inhibitory or activating effect on T cells, SeLe30 increased the production of IL-6 and IL-10, which suggested its influence on peripheral blood monocytes/macrophages as well [43]. Therefore, the use of this model limited the ability to describe the direct effects of SeLe30 on T cells.

To distinguish the indirect effects of SeLe30 on T cells through the action of other immune cells, in the present study, we conducted analyses on isolated CD3+ T cells while simultaneously evaluating its effect on PBMC cultures. Moreover, we assessed the effect of SeLe30 on the expression of CD25, CD366 (TIM-3), and CD279 (PD-1) markers; the subsets of CD8+ T cells; and IFN-γ, IL-6, and TNF-α production.

CD25 is the α-chain of the trimeric IL-2 receptor and a middle/late cellular activation marker of mainly T cells, B cells, and NK cells. It is expressed on the surface of T cells approximately 24–48 h after stimulation of the T-cell receptor (TCR)/CD3 complex and remains high after 4–5 days [44,45]. CD279, more commonly known as PD-1, is a coinhibitory receptor expressed during activation by all T cells to regulate its effector functions during various physiological responses and homeostasis [46]. Although it plays an important physiological role in balancing protective immunity and tolerance, during a chronic response to pathogens or cancer, PD1 expression may limit protective immunity and is considered a marker of exhausted T cells. Exhausted T cells do not proliferate and are characterized by low effector functions, such as cytotoxicity and cytokine production [45]. Another marker that is upregulated on exhausted T cells is CD366 (TIM-3), which is involved in CD8^+^ T-cell depletion. Its interactions with ligands (galectin-9, carcinoembryonic antigen-related cell adhesion molecule 1 (biliary glycoprotein) (CEACAM1), and high-mobility group protein B1 (HMGB1)) mediate its inhibitory function. Therefore, both CD279 and CD366 function as negative regulators of T-cell responses [47].

Our study demonstrated that SeLe30 downregulated the expression of CD25, CD279, and CD366 on CD4+ and CD8+ T cells stimulated by anti-CD3 Ab. Conversely, it upregulated their expression on unstimulated isolated CD4+ and CD8+ T cells, as well as those stimulated with anti-CD3/CD28 Abs. Regardless of whether the effect of SeLe30 on isolated T cells or PBMCs was analyzed, a similar direction of action was demonstrated. However, the effect was statistically more pronounced in the case of PBMC cultures, which may suggest that it was also shaped by T-cell interactions with other immune cells. Both anti-CD3 and anti-CD3/CD28 Abs are frequently used in T-cell proliferation protocols. Anti-CD3 Ab delivers a strong proliferative signal through the TCR; however, in the absence of an additional costimulatory signal, proliferation is followed by premature apoptosis or anergy [48]. It was documented that CD4+ T cells respond very well to anti-CD3/CD28 stimulation, but the single anti-CD3 Ab has greater potential to upregulate CD8+ T-cell proliferation [49]. As mentioned above, numerous immune system receptors interact in important ways with fungal α- and β-glucans. Comer et al. [50] reported that β-1,3 glucans specifically bind to CD28 and stimulate T-cell activation in collaboration with anti-CD stimulation. Wagner and colleagues [51] found that blocking the CD11b subunit of CD3 results in the inhibition of T-cell proliferation and activation after anti-CD3 Ab stimulation [50]. Therefore, we hypothesize that SeLe30 may block CR3 and thus downregulate CD25, CD279, and CD366 expression in T cells stimulated only with anti-CD3 Ab. There is still little research on fungal α- and β-glucans’ influence on CD25, CD279, and CD366 expression. Similar to our results, Wang et al. reported that lentinan, a -1,3-branched-1,6-D-glucan, downregulated the number of CD4+CD25+ T cells in a murine model of sepsis induced by *P. aeruginosa* infection [14]. In contrast, the group found that this drug increased the number of CD4+CD25+ T cells in patients with non-small-cell lung cancer treated with vinorelbine and cisplatin [23]. Accordingly, a study by Wu et al. showed that lentinan effectively upregulated CD4+CD25+Foxp3+ regulatory T cells in the spleen, pancreatic lymph nodes, and pancreas of non-obese diabetic (NOD) mice [52].

In the present study, we have found that in the PBMC culture stimulated with the anti-CD3 Ab, SeLe30 increased the percentage of naïve and central memory cells, whereas the number of effector memory T cells increased when stimulated with anti-CD3/CD28 Abs. We assume that in the CD3/CD28 activation model, SeLe30 promotes the differentiation of CD8+ T cells toward effector memory T cells, with a high proliferative potential, which may partially explain the increased proliferation in this model. It should also be emphasized that the polysaccharide did not influence the change in the above-mentioned subpopulations in the culture of isolated CD3+ T cells in comparison with the observed effect on the PBMC culture.

In a study conducted in mice, Kajiyama et al. [53] isolated CD8+ T cells from spleens and co-cultured them with lentinan under stimulation with anti-CD3 Ab. The group demonstrated that this drug increased the number of CD8+ effector memory T cells. Xu et al. [54] also analyzed the effect of lentinan on CD8+ T-cell subpopulations and revealed that this drug elevated the percentage of CD8+ central memory T cells in LAP-R1 and LAP-L1 lung tumors in mice.

The aim of our study was also to evaluate and compare SeLe30’s impact on the production of IFN-γ, IL-6, and TNF-α by isolated CD3+ T cells versus PBMCs. These proinflammatory cytokines were selected for this study because it has been proven that Se, on a cellular level, influences cytokine secretion. Moreover, our preliminary analyses suggested that SeLe30 may have a direct effect on the activation of the TCR/CD3 complex in T cells, which may result in the regulation of IFN-γ, IL-6, and TNF-α production.

The effects of IFN-γ, IL-6, and TNF-α on immune cells are context-dependent and may vary under homeostatic or disease conditions. But, overall, they are critical regulators of the immune response and the development and proper functioning of the immune system. While IFN-γ enhances antigen presentation, antimicrobial responses, and chemokine production and decreases the proliferation of immune cells [55], TNF-α mediates signaling for cell survival or cell death [56]. IL-6 is the most ambiguous of these cytokines, playing central roles in immune response activation and modulation, being able to act in a proinflammatory or anti-inflammatory manner, and physiologically participating in the differentiation of various immune cells [57]. We found that SeLe30 increased the production of these cytokines; however, the observed effect was stronger in PBMC cultures. The obtained results suggest that SeLe30 not only has a clear effect on modulating the immune response via T cells but also has an impact on other immune cell populations. Many studies have examined the effects of *L. edodes*-derived polysaccharides on cytokine levels in healthy humans, patients, and animal models; however, some of these studies reported opposite results. Wang et al. [23], similar to our study, demonstrated that chemo-immunotherapy (vinorelbine, cisplatin, and lentinan) resulted in upregulated IFN-γ and TNF-α production in patients with non-small-cell lung cancer. Consistent results were obtained in a study by Tanigawa et al. [58], who reported elevated IFN-γ production by PBMCs of cancer patients treated with immunotherapy and *L. edodes* mycelia extract (named LEM). Another *L. edodes*-isolated bioactive compound containing α-1,4-glucans (named AHCC^®^), similar to SeLe30, increased the secretion of IFN-γ and TNF-α by T cells of healthy adults [59]. In a randomized, double-blind, placebo-controlled study, the effect of rice bran fermented with *L. edodes* mycelia (containing polysaccharides) on serum IFN-γ and TNF-α was analyzed in healthy adults [60]. It was found that such supplementation significantly increased IFN-γ production compared with the placebo group, without an effect on TNF-α. In contrast to our findings, Zembron-Lacny and colleagues demonstrated that *L. edodes* extract did not affect IL-6 and TNF-α serum levels in healthy men exposed to exercise-induced skeletal muscle damage [61]. Morales et al. [62] evaluated the immunomodulatory effect of a β-D-glucan-enriched (BGE) extract obtained from *L. edodes* in patients with untreated mild hypercholesterolemia. After eight weeks, no significant changes in IL-6 and TNF-α production were found. In another double-blind, placebo-controlled study, the effect of a β-glucan from *L. edodes* mycelium (named Lentinex) was analyzed in healthy elderly subjects, and the authors likewise reported no effect on cytokine secretion [22]. The aim of another randomized study was to determine whether the consumption of whole, dried L. edodes affects the immune system. It was demonstrated that consuming the mushrooms upregulated, among others, TNF-α, but had no effect on IL-6 and IFN-γ [63].

The overwhelming majority of studies have demonstrated that various *L. edodes* polysaccharides exert immunomodulatory effects, both in humans and in animal models. However, the observed contradictory outcomes may result from the polysaccharide source, the methods of mushroom cultivation and extraction, and their composition, as well as the route of administration.

Isolated from the Se-enriched *L. edodes* mycelium SeLe30 fraction, a mixture of linear 1,4-α-glucan and linear 1,3-β- and 1,6-β-glucans exerts immunomodulatory effects on human T cells. Its biological activity is context-dependent.

Our results indicate that SeLe30 exhibits an enhancing effect on the regulatory potential of the immune response. On the one hand, it acts on unstimulated T cells, affecting their activation while increasing the expression of inhibitory molecules (immune checkpoints), which sensitizes them to inhibitory signals that can silence this activation. In the case of cells stimulated by an insufficient signal (lack of costimulation), SeLe30 exhibits an inhibitory effect, reducing their activation. On the other hand, in the case of cells stimulated by a strong, dual signal, its effect is further enhanced, again increasing the “safety brake” of the PD1 and TIM3 inhibitory molecules. However, the final effect of SeLe30 is mediated by its indirect effects by altering interactions with other immune cells, as evidenced by the differences between the results on the isolated T-cell fraction and PBMCs. In addition, by upregulating the production of IL-6, IFN-γ, and TNF-α, SeLe30 clearly affects the modulation of the immune response by mobilizing the immune system for protection. Finally, our results may suggest a direct effect on the activation of CD3-dependent signaling proteins. Further studies, particularly the analysis of the effect of SeLe30 on TCR/CD3 complex activation, are necessary to fully explain the mechanism of its biological activity.

## 4. Materials and Methods

### 4.1. Biosynthesis and Isolation of SeLe30

SeLe30 biosynthesis, isolation, and structural analysis were described in detail in our previous paper [42]. In brief, the *L. edodes* (Berk.) Pegler strain used in this study was American Type Culture Collection 48085 (ATCC, Manassas, VA, USA). Sodium selenite (Sigma, Saint Louis, MO, USA) was added to the culture medium to a final concentration of 30 μg/mL. *L. edodes* mycelium, after cultivation for 10 days at 26 °C, was harvested by filtration, washed, and freeze-dried. SeLe30 was isolated by the modified Chihara method [39,40]. The endotoxin level, determined by Limulus amebocyte lysate (LAL), was less than 0.01 EU/µg of SeLe30. Structural studies have shown that the fraction is a mixture of selenium-containing polysaccharides: α-1,4-D-glucan (molecular weight 2.25 × 10^6^ g/mol), unbranched β-1,6-D-glucan, unbranched β-1,3-D-glucan, and β-1,3-branched β-1,6-D-glucan (molecular weight 1.10 × 10^5^ g/mol). Selenium in polysaccharide molecules is present in Se-glycosidic bonds [39].

### 4.2. PBMCs and CD3^+^ T-Cell Isolation

Peripheral blood mononuclear cells (PBMCs) were isolated from the buffy coats of 22 healthy anonymous donors (age 18–64 years) that were obtained from the Regional Blood Donation and Blood Treatment Center. Buffy coats were diluted 1:2 in PBS in 50 mL Falcon tubes. PBMCs were isolated by density gradient centrifugation on Histopaque-1077 (Sigma-Aldrich, Saint Louis, MO, USA) according to manufacturer instructions. PBMCs were collected from a plasma/Histopaque-1077 interface with a Pasteur pipette and transferred to a new 50 mL Falcon tube. Isolated PBMCs were then washed four times in PBS to rinse the cell pellets and to reduce platelet contamination. Finally, cells were suspended in Roswell Park Memorial Institute (RPMI) 1640 medium (Gibco, Thermo Fisher Scientific, Waltham, MA, USA) with 10% heat-inactivated fetal bovine serum (Sigma, Saint Louis, MO, USA) and 1% penicillin–streptomycin (Biowest, Riverside, MO, USA). After determining the number of PBMCs, 1 × 10^7^ cells were collected for CD3+ T-cell isolation by a MojoSort^TM^ Human CD3 T Cell Isolation Kit (BioLegend, San Diego, CA, USA) [64]. The purity of isolated CD3+ T cells was above 96%, as examined by flow cytometry.

PBMCs and CD3+ T cells were used to assess the effect of SeLe30 on the profile of secreted cytokines, immune checkpoints (ICPs), activation marker expression on CD4+ and CD8+ T cells, and CD8+ T-cell phenotype.

This study was approved by the Bioethics Committee of the Medical University of Warsaw (no. KB/174/2017; updated AKBE/186/2021).

### 4.3. Flow Cytometry for Expression of ICPs, Activation Marker on T Cells, and CD8 T-Cell Subsets

PBMCs and CD3+ T cells (2 × 10^5^ cells/well) were seeded on flat-bottom 96-well plates (Greiner Bio-One Kremsmünster, Kremsmünster, Austria) and cultured for 24 h in the following variants: (1) unstimulated cells; (2) cells stimulated with anti-CD3/CD28 Abs (ratio 2:5, Gibco, Waltham, MA, USA); (3) cells stimulated anti-CD3 Ab (coated on plate wells, 0.75 µg/mL, BD Pharmingen, Franklin Lakes, NJ, USA); (4) unstimulated cells incubated in the presence of SeLe30 (100 µg/mL); (5) anti-CD3/CD28-stimulated cells incubated in the presence of SeLe30 (100 µg/mL); (6) anti-CD3-stimulated cells incubated in the presence of SeLe30 (100 µg/mL). Each experiment was performed in triplicate.

Prior to flow cytometry analyses, all antibodies were titrated to obtain the highest signal-to-noise ratio for each fluorochrome. Fluorescence-Minus-One experiments were performed in order to determine the cut-off value for the positive population for each marker [65].

Cells were harvested after 24 h of culture. Beads coated with anti-CD3/CD28 Abs were removed in a magnetic field. Next, cells were washed in Stain Buffer (BSA, BD Biosciences, San Jose, CA, USA) and stained with fluorescent antibodies: anti-CD3 PerCP (clone SK7, BD Biosciences, San Jose, CA, USA), anti-CD25 FITC (clone 2A3, BD Biosciences, San Jose, CA, USA), anti-CD8 APC (clone SK1, BD Biosciences, San Jose, CA, USA), anti-CD4 APC-Cy7 (clone SK3, BD Biosciences, San Jose, CA, USA), anti-CD279 BV480 (clone EH12.1, Becton Dickinson, Franklin Lakes, NJ, USA), anti-CD366 PE (clone 7D3, Becton Dickinson, NJ, USA), anti-CD45RO BV711 (clone UCHL-1, Becton Dickinson, NJ, USA), and anti-CD62L (clone DREG-56, Becton Dickinson, NJ, USA). Cells were stained for 15 min at room temperature in the dark and resuspended in 100 µL of Stain Buffer (BSA, BD Biosciences, San Jose, CA, USA). In each experiment, samples were analyzed for singlet events with doublet discrimination. Gating on CD3+ cells identified T cells, gating on CD4+ cells identified T helper (Th) cells, and gating on CD8+ cells identified T cytotoxic (Tc) cells. CD4+ and CD8+ T cells were subsequently analyzed separately for the expression of CD25, CD279 (PD-1), and CD366 (Tim-3). The percentage of central memory lymphocytes (CD45RO+CD62L− phenotype), effector memory lymphocytes (CD45RO+CD62L+ phenotype), and naïve lymphocytes (CD45RO−CD62L+ phenotype) was also determined, and the effect of SeLe30 on changes in these populations was analyzed.

All samples were run on a CytoFlex flow cytometer (Beckman Coulter, Brea, CA, USA), and data were analyzed with CytExpert software, version 2.4 (Beckman Coulter, Brea, CA, USA).

### 4.4. Quantification of IFN-γ, IL-6, and TNF-α Concentrations

Cell culture supernatants were aspirated from plates, centrifuged, and stored at −80 °C until further analysis. Cytokine concentrations were assessed using a MILLIPLEX^®^ Human Cyto Panel A (Merck Millipore, Darmstadt, Germany) and Luminex 200 analyzer instrument (Merck Millipore, Darmstadt, Germany) [66]. Before analysis, instrument calibration and verification were performed using the MAGPIX Calibration Kit and MAGPIX Performance Verification Kit (Merck Millipore, Darmstadt, Germany). A panel of three cytokines (IFN-γ, IL-6, and TNF-α) was selected for analysis and measured according to the manufacturer’s protocol. Briefly, 25 µL of supernatant was incubated with 25 µL of antibody-coated microparticles for 2 h at room temperature. After washing (3× with 200 µL of Wash Buffer), samples were incubated for 1 h at room temperature with 25 µL of biotinylated Abs. Following a wash (3× with 200 µL of Wash Buffer), 25 µL of streptavidin–phycoerythrin conjugate was added. After the final wash (3× with 200 µL of Wash Buffer), the microparticles were resuspended in 150 µL of the assay buffer and analyzed. The assay was performed in duplicate.

### 4.5. Cell Viability Assay

PBMCs and CD3+ T cells (2 × 10^5^ cells/well) were seeded on flat-bottom 96-well plates (Greiner Bio-One Kremsmünster, Austria) and cultured for 24 h at 37 °C in a humidified atmosphere with 5% CO_2_ in the presence of SeLe30 (100 µg/mL) and with an equivalent amount of medium and water for injection as controls. After incubation, cells were harvested, washed in 2 mL of PBS, resuspended in 100 µL of Zombie Violet™ (BioLegend, San Diego, CA, USA) at a ratio of 1:400, incubated for 20 min, washed with 2 mL of Stain Buffer, and labeled with anti-CD3-PerCP AB (clone SK7, BD Biosciences, San Jose, CA, USA) in 100 µL of Stain Buffer for 15 min. Next, cells were washed in 2 mL of Stain Buffer, resuspended in 100 µL of PBS with 0.01% sodium aide, and acquired with a DxFlex flow cytometer. For each variant, the percentage of CD3+ T lymphocytes positive for Zombie Violet dye was recorded and compared to control cultures.

### 4.6. Statistical Analysis

Statistical analysis and data visualization were performed using GraphPad Prism 9.4.0 (GraphPad Software). The normality of the data set distribution was tested using Kolmogorov–Smirnov, Shapiro–Wilk, Anderson–Darlin, D’Agostino, and Pearson tests. The data set was considered to have a normal distribution when each of the applied tests had a prediction value higher than 0.05. The determination of outliers was performed using both ROUT (Q = 1%) and Grubbs’ (α = 0.05) methods. The Student *t*-test was performed when the distribution of differences was normal, and the Wilcoxon test was used when the distribution of differences was not normal. A *p*-value of <0.05 (*) was considered statistically significant, and *p* < 0.01 (**) or *p* < 0.001 (***) was highly significant. Graphs are presented as mean ± SEM (standard error of the mean).

## Data Availability

The data presented in this study are available on request from the corresponding author without any restrictions.
